# Acetaminophen use during pregnancy and DNA methylation in the placenta of the extremely low gestational age newborn (ELGAN) cohort

**DOI:** 10.1093/eep/dvz010

**Published:** 2019-08-06

**Authors:** Kezia A Addo, Catherine Bulka, Radhika Dhingra, Hudson P Santos, Lisa Smeester, T Michael O’Shea, Rebecca C Fry

**Affiliations:** 1 Curriculum in Toxicology and Environmental Medicine, School of Medicine, University of North Carolina, Chapel Hill, NC, USA; 2 Department of Environmental Sciences and Engineering, Gilling School of Global Public Health, University of North Carolina, Chapel Hill, NC, USA; 3 Institute for Environmental Health Solutions, Gilling School of Global Public Health, University of North Carolina, Chapel Hill, NC, USA; 4 School of Nursing, University of North Carolina, Chapel Hill, NC, USA; 5 Department of Pediatrics, School of Medicine, University of North Carolina, Chapel Hill, NC, USA

**Keywords:** placenta, DNA methylation, sexual dimorphism, acetaminophen, prenatal, maternal, endocrine disruption

## Abstract

Acetaminophen is considered the safest antipyretic and analgesic medication for pregnant women. However, studies have reported that acetaminophen has endocrine disrupting properties and prenatal exposure has been associated with early life epigenetic changes and later life health outcomes. As the placenta is the central mediator of maternal and fetal interactions, exposure to acetaminophen during pregnancy could manifest as perturbations in the placenta epigenome. Here, we evaluated epigenome-wide cytosine-guanine dinucleotide (CpG) methylation in placental tissue in relation to maternal acetaminophen use during pregnancy in a cohort of 286 newborns born prior to 28 weeks gestation. According to maternal self-report, more than half (166 of 286) of the newborns were exposed to acetaminophen *in utero*. After adjustment for potential confounders, a total of 42 CpGs were identified to be differentially methylated at a false discovery rate < 0.05, with most displaying increased methylation as it relates to acetaminophen exposure. A notable gene that was significantly associated with acetaminophen is the prostaglandin receptor (*PTGDR*) which plays an essential role in mediating placental blood flow and fetal growth. Moreover, for 6 of the 42 CpGs, associations of acetaminophen use with methylation were significantly different between male and female placentas; 3 CpG sites were associated with acetaminophen use in the male placenta and 3 different sites were associated with acetaminophen use in the female placenta (*P*_interaction_ < 0.2). These findings highlight a relationship between maternal acetaminophen use during pregnancy and the placental epigenome and suggest that the responses for some CpG sites are sex dependent.

## Introduction

Acetaminophen (*N*-actyl-4-aminophenol), also known as paracetamol, is one of the most widely used analgesic and antipyretic drugs in the world [1, 2]. Nearly two-thirds of pregnant women in the USA and more than half of pregnant women across Europe report taking acetaminophen [1, 3, 4]. Acetaminophen is classified as a Category B drug (i.e. no risks observed in pregnant women) by the U.S. Food and Drug Administration [4]. Still, acetaminophen and its metabolites freely cross the placenta and have been found in cord blood, newborn urine, and fetal liver, suggesting the potential for direct fetal toxicity [5–7]. 

Several epidemiologic studies have observed associations between prenatal acetaminophen exposure with adverse birth and later life health outcomes. For example, prenatal acetaminophen use has been linked to low birth weight, birth defects, miscarriages, and preterm birth [8, 9]. In addition to the early life outcomes, later in life effects have been observed as well including neurodevelopmental disorders such as attention deficit-hyperactivity disorder (ADHD)/hyperkinetic disorder and autism spectrum disorder (ASD) [10–13]. Furthermore, acetaminophen use in pregnancy has also been associated with an increased risk of cryptorchidism and hypospadias in male infants [14]. Given these findings, more research is needed to characterize the potential underlying molecular mechanisms by which prenatal acetaminophen exposure may influence childhood health.

The latency of the adverse health outcomes highlighted in the above epidemiologic studies is suggestive of fetal epigenetic programming. Xenobiotic exposures during critical windows of development have been shown to program the fetal epigenome, likely through maternal–fetal interaction via the placenta, leading to permanent biological and physiological change [15–17]. Among the molecular mechanisms by which the epigenome can be modified, DNA methylation of cytosine-guanine dinucleotides (CpGs) is a major process by which the placenta dynamically responds to changing conditions throughout pregnancy [17–19]. For example, in the context of environmental contaminants, several studies have associated *in utero* metals exposure to CpG methylation in the placenta [17, 18, 20, 21]. In support of the potential for acetaminophen to impact the epigenome, a recent study has demonstrated an association between prenatal acetaminophen exposure and CpG methylation in banked cord blood of children with ADHD [22]. Importantly, DNA methylation may be used as biomarker of *in utero* exposure and a predictor of later life outcomes [23]. 

The placenta is the master regulator of the fetal environment because it mediates the exchange of nutrient and gas exchange, and also dynamically responds to the changing conditions throughout pregnancy [24, 25]. Studies in both rodents and humans have demonstrated that a wide range of maternal insults such as diet, smoking, alcohol intake, and drug exposure can elicit marked transformation in placental physiology, morphology, epigenome, and gene expression which can impair both fetal development and have long-term impacts on the offspring health [26–28]. In relation to acetaminophen, studies have shown that acetaminophen impacts placental trophoblast cells where it induces oxidative stress and downregulates the expression of the placental efflux pump [29]. Specifically, acetaminophen reduces both mRNA and protein expression of ATP-binding cassette super-family G2 (ABCG2) in human trophoblast cells and decreases total glutathione levels in rat placentas [29]. For these reasons, the placenta may be an ideal tissue to investigate fetal programming and the impact of *in utero* acetaminophen exposure.

In addition to mediating maternal–fetal interactions, the placenta is an intriguing organ known to display sex-based differences in infant health outcomes and responses to perinatal stressors [30]. This has been observed in both humans and rodents at the level of the placental DNA methylome and transcriptome [15, 30, 31]. In addition to the sexually dimorphic response of the placenta to *in utero* exposures, epidemiologic studies have associated prenatal acetaminophen with adverse birth outcomes disproportionately affecting males [32]. In rodents, several studies have reported sex-related differences in susceptibility to acetaminophen hepatotoxicity, where females show greater resistances to acetaminophen-induced liver injuries than males [33–35].

In the present study, we set out to assess the relationship between prenatal acetaminophen use and the placental epigenome derived from 286 singleton newborns of the Extremely Low Gestational Age Newborns (ELGANs) study and to determine whether the relationship could be modified by infant sex. It was hypothesized that maternal acetaminophen use during pregnancy would be associated with differential CpG methylation profiles in placental tissue in a sexually dimorphic manner. To our knowledge, this study presents the first genome-wide analysis of placental DNA methylation as it relates to acetaminophen exposure *in utero*.

## Results

### Participant Characteristics

A total of 286 singletons (94% of the *n* = 305 subsample with placental methylation data available) were included in the analyses after excluding 19 newborns who were missing maternal data regarding acetaminophen use in pregnancy (*n* = 13), race/ethnicity (*n* = 2), or prepregnancy body mass index (*n* = 4). Of these, 166 (58%) mothers self-reported using acetaminophen at least once during their pregnancy. Descriptive statistics for mothers and their newborns are presented by acetaminophen use status in Table 1. Mothers who reported using acetaminophen while pregnant were generally similar to those who did not, with some notable exceptions. Non-Hispanic white mothers tended to use acetaminophen more than their non-Hispanic black, non-Hispanic other, or Hispanic counterparts. Mothers who reported acetaminophen use had experienced higher rates of acute illnesses during pregnancy. The majority of mothers who reported using acetaminophen while pregnant used the drug at least once in each trimester (102 of 166, data not shown). Newborn characteristics, including sex, gestational age, and birth weight, were similar between mothers who did and did not use acetaminophen in pregnancy.

**Table 1:** maternal and newborn characteristics by self-reported acetaminophen use during pregnancy within the ELGAN cohort

			Male newborns		Female newborns	
Characteristic	No acetaminophen (*n* = 120)	Acetaminophen (*n* = 166)	No acetaminophen (*n* = 63)	Acetaminophen (*n* = 88)	No acetaminophen (*n* = 57)	Acetaminophen (*n* = 78)
*Maternal*						
Age (years), mean ± SD	28 ± 7	29 ± 6	29 ± 8	28 ± 6	28 ± 7	29 ± 7
Race/ethnicity, %						
Non-Hispanic white	40.8	63.9	38.1	65.9	43.9	61.5
Non-Hispanic black	5.0	1.8	3.2	1.1	7.0	2.5
Non-Hispanic other	41.7	22.9	46.0	21.6	36.8	24.4
Hispanic	12.5	11.5	12.7	11.4	12.3	11.5
Educational attainment, %						
High school diploma or less	47.5	39.8	46.0	43.2	49.1	35.9
At least some college	20.0	26.5	23.8	25.0	15.8	28.2
College degree or greater	32.5	33.7	30.2	31.8	35.1	35.9
Publicly insured, %	39.2	34.3	38.1	35.2	40.4	33.3
Cigarette smoke^a^ exposure while pregnant, %	23.3	31.9	17.5	35.2	29.8	28.2
Prepregnancy BMI, mean ± SD	26.9 ± 7.8	25.5 ± 7.1	27.7 ± 8.4	26.9 ± 7.8	26.1 ± 7.2	25.5 ± 6.6
Acutely ill while pregnant, %	29.2	42.2	27.0	37.5	31.6	47.4
Chronically ill while pregnant, %	17.5	17.5	15.9	15.9	19.3	19.2
NSAID use while pregnant, %	13.3	12.7	17.5	10.2	8.8	15.4
Parity, %						
1	60.8	51.2	55.6	50.0	66.7	52.6
2–3	31.7	39.2	36.5	40.9	26.3	37.2
4+	7.5	9.6	7.9	9.1	7.0	10.3
*Delivery*						
State of occurrence, %						
Connecticut	5.0	7.2	6.4	9.1	3.5	5.1
Illinois	12.5	6.6	17.5	4.6	7.0	9.0
Massachusetts	34.2	31.3	38.1	27.3	29.8	35.9
Michigan	14.2	25.9	9.5	28.4	19.3	23.1
North Carolina	34.2	28.9	28.6	30.7	40.4	26.9
Type, %						
Caesarian	64.2	60.2	60.3	60.2	68.4	60.3
Vaginal	35.8	39.8	39.7	39.8	31.6	39.7
*Newborn*						
Gestational age (weeks), mean ± SD	25.9 ± 1.3	25.9 ± 1.3	26.0 ± 1.2	25.8 ± 1.3	25.9 ± 1.3	26.0 ± 1.3
Birth weight (g), mean ± SD	836.9 ± 205.4	824.1 ± 184.1	846.0 ± 207.4	841.1 ± 179.8	826.7 ± 204.5	804.9 ± 188.0
Sex, *n* %						
Male	52.5	53.0	100.0	100.0		
Female	47.5	47.0			100.0	100.0

SD, standard deviation.

a Active or passive (secondhand) exposure.

### Differentially Methylated CpGs Associated with Acetaminophen Exposure

**Figure 1:**
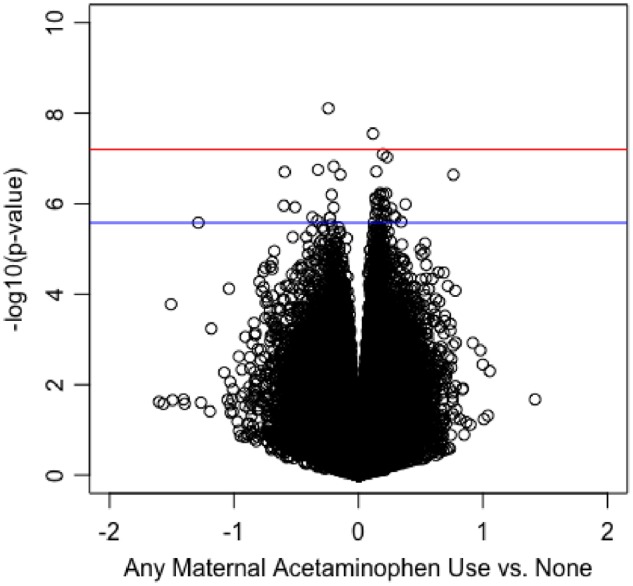
volcano plot illustrating the relationship between maternal acetaminophen use during pregnancy compared to no use with methylation across 790 677 CpGs, adjusted for maternal age, race/ethnicity, educational attainment, public health insurance status, cigarette smoke exposure, prepregnancy body mass index, maternal acute illness, maternal chronic illness, maternal NSAID use, parity, newborn sex, gestational age, birth weight, and putative cell type proportions. The blue line represents the false discovery rate (*q* < 0.05). The red line represents a Bonferroni correction of 6.3 × 10^−8^

The multivariable model results for associations between acetaminophen use during pregnancy and placental CpG methylation showed evidence of genomic inflation (*λ* = 1.54, Supplementary Fig. S2), which can be indicative of a high number of false positives. We therefore applied *bacon* correction to the model coefficients and corresponding *P* values [36]. This procedure reduced the inflation factor to 1.08 (Supplementary Fig. S2). In the *bacon-*corrected multivariable models that adjusted for potential confounders including putative cell types, a total of 42 differentially methylated CpG probes were identified in relation to maternal acetaminophen use based on a false discovery rate <0.05 (Table 2). Two of these 42 CpGs reached Bonferroni significance at 6.3 × 10^−8^. These were cg12202498 located in the 3′UTR of ELL Associated Factor (*EAF1*) and cg09643313 located in the promoter region of Protein phosphatase 9A (*PPP1R9A*). Effect estimates and corresponding *P* values for acetaminophen use were plotted (Fig. 1), with the majority (*n* = 28, 66.7%) of significant probes displaying increased methylation. A Manhattan plot showing the genomic distribution of associations is provided (see Supplementary Fig. S3**)**. In addition, distributions of the methylation *β* values for the 42 differentially methylated sites are presented in Supplementary Fig. S4. Thirteen of the significant probes were located in intergenic regions, with the remainder mapping to 29 genes. Although no single gene contained more than one differentially methylated CpG, two probes (cg05912084 and cg09351315) were located within 10 bp of each other in an intergenic region of chromosome 17 between *CBX8* and *CBX2*. There was significant over-representation of differentially methylated within north shelf regions (i.e. 2–4 kb north of a CpG island; *P* = 5.20E–05, see Supplementary Fig. S5). Results for all 790 677 probes in relation to acetaminophen use during pregnancy are displayed in Supplementary Table S2.

**Table 2:** EWAS results of any maternal acetaminophen use during pregnancy among ELGAN singletons (*n* = 286), sorted by chromosome

					No acetaminophen (n = 120)	Acetaminophen (n = 166)				
CpG probe	Gene	Chr: position^a^	Feature category^b^	Relation to CpG island	Median *β* value	Median *β* value	Coefficient^c^	SE^c^^,d^	*P* value^c^	*q* value^c^
cg26893147		1: 153493755	Intergenic	Open Sea	0.843	0.864	0.221	0.046	1.45E–06	3.95E–02
cg13442152	*DCAF6*	1: 168044891	3'UTR	Open Sea	0.961	0.963	0.165	0.034	8.25E–07	3.62E–02
cg12244031		2: 37559170	Intergenic	Open Sea	0.979	0.981	0.138	0.029	1.73E–06	4.27E–02
cg02598079		2: 64834106	Intergenic	North Shelf	0.138	0.135	–0.231	0.049	2.06E–06	4.41E–02
cg22974428		2: 183737450	Intergenic	Open Sea	0.953	0.959	0.197	0.040	6.14E–07	3.45E–02
cg09050313	*MOBKL3*	2: 198380581	5'UTR; TSS200	Island	0.010	0.009	–0.216	0.043	6.33E–07	3.45E–02
cg00235870	*BCS1L*	2: 219526577	Body	South Shelf	0.952	0.956	0.151	0.031	1.45E–06	3.95E–02
cg12202498	*EAF1*	3: 15482605	3'UTR	Open Sea	0.739	0.755	0.117	0.021	2.83E–08	1.12E–02
cg18772158	*GALNTL2*	3: 16215032	TSS1500	Open Sea	0.425	0.475	0.343	0.073	2.50E–06	4.94E–02
cg18517818		4: 10956577	Intergenic	Open Sea	0.191	0.154	–0.509	0.105	1.19E–06	3.83E–02
cg23038580	*FAM47E*	4: 77184168	Body	Open Sea	0.956	0.958	0.158	0.033	1.32E–06	3.85E–02
cg07345722		4: 94731982	Intergenic	Open Sea	0.019	0.013	–0.592	0.114	1.98E–07	1.81E–02
cg21956099	*TERT*	5: 1278018	Body	South Shore	0.787	0.800	0.210	0.043	1.12E–06	3.83E–02
cg23956317	*GPR98*	5: 89967376	Body	Open Sea	0.717	0.742	0.378	0.077	1.03E–06	3.83E–02
cg19670286	*GFRA3*	5: 137610127	1stExon; 5'UTR	Island	0.007	0.004	–1.286	0.274	2.63E–06	4.95E–02
cg16301827	*BTNL9*	5: 180477462	Body	North Shelf	0.737	0.805	0.762	0.147	2.29E–07	1.81E–02
cg01607625	*PPP1R2P1*	6: 32847830	Body	Island	0.123	0.114	–0.599	0.123	1.10E–06	3.83E–02
cg10970399	*USP45*	6: 99963512	TSS1500	South Shore	0.007	0.006	–0.228	0.048	2.04E–06	4.41E–02
cg10552522		6: 167342122	Intergenic	Open Sea	0.955	0.960	0.184	0.039	2.26E–06	4.71E–02
cg07825336	*SMOC2*	6: 169039836	Body	Open Sea	0.965	0.969	0.286	0.060	1.86E–06	4.41E–02
cg09643313	*PPP1R9A*	7: 94537893	TSS1500; 5'UTR	South Shore	0.010	0.008	–0.242	0.042	7.79E–09	6.16E–03
cg13947310	*PLXNA4*	7: 132123957	Body	Open Sea	0.744	0.705	–0.330	0.070	2.36E–06	4.79E–02
cg27569423		8: 23138893	Intergenic	Open Sea	0.129	0.145	0.209	0.043	1.05E–06	3.83E–02
cg15551981		8: 28552181	Intergenic	Open Sea	0.383	0.358	–0.198	0.038	1.49E–07	1.81E–02
cg14279752	*MTSS1*	8: 125573314	Body	Open Sea	0.973	0.975	0.144	0.028	1.95E–07	1.81E–02
cg03242028	*MSMP*	9: 35754421	TSS200	North Shelf	0.882	0.889	0.126	0.027	2.59E–06	4.95E–02
cg12944204	*MRPL23*	11: 1971969	Body	North Shelf	0.940	0.946	0.262	0.054	1.26E–06	3.83E–02
cg06912304	*STIM1*	11: 4113452	3'UTR	North Shelf	0.801	0.798	0.145	0.030	9.86E–07	3.83E–02
cg12176456	*RCN1*	11: 32101922	Body	Open Sea	0.953	0.958	0.179	0.037	1.52E–06	3.96E–02
cg20074591	*KCNE3*	11: 74179549	TSS1500	South Shore	0.913	0.923	0.300	0.063	1.99E–06	4.41E–02
cg05554891	*FAM186A*	12: 50790101	1st Exon	Open Sea	0.979	0.981	0.137	0.028	7.47E–07	3.62E–02
cg11178884	*MARS*	12: 57906632	Body	Open Sea	0.966	0.970	0.170	0.034	6.55E–07	3.45E–02
cg14316800	*PSMD9*	12: 122352951	Body	North Shelf	0.957	0.960	0.139	0.028	7.90E–07	3.62E–02
cg02191312	*PTGDR*	14: 52734397	TSS200	Island	0.236	0.212	–0.325	0.062	1.78E–07	1.81E–02
cg20648668		17: 17279942	Intergenic	Open Sea	0.659	0.680	0.196	0.036	8.12E–08	1.81E–02
cg09351315		17: 77775823	Intergenic	Island	0.622	0.635	0.175	0.035	5.89E–07	3.45E–02
cg05912084		17: 77775833	Intergenic	Island	0.527	0.539	0.229	0.043	9.34E–08	1.81E–02
cg00700039	*SMCHD1*	18: 2659555	Body	South Shelf	0.676	0.667	–0.199	0.041	1.21E–06	3.83E–02
cg06404695		18: 76148311	Intergenic	North Shelf	0.179	0.149	–0.369	0.078	2.00E–06	4.41E–02
cg16412670	*ATP9B*	18: 76985542	Body	Island	0.965	0.969	0.224	0.045	5.96E–07	3.45E–02
cg24476584	*ZNF837*	19: 58883830	5'UTR	South Shelf	0.276	0.266	–0.146	0.028	2.27E–07	1.81E–02
cg10629004	*PAX1*	20: 21696467	3'UTR	South Shore	0.877	0.890	0.201	0.042	1.55E–06	3.96E–02

a Chr, chromosome.

b Gene feature category of the methylation probe; TSS, transcription start site; TSS200, 200 bases from TSS; TSS1500, 1500 bases from TSS; UTR, untranslated region.

c Linear model results of methylation *M* values regressed on any maternal acetaminophen use during pregnancy compared to no use, adjusted for maternal age, race/ethnicity, educational attainment, public health insurance status, cigarette smoke exposure, prepregnancy body mass index, maternal acute illness, maternal chronic illness, maternal NSAID use, parity, newborn sex, gestational age, birth weight, and putative cell type proportions.

d SE, standard error.

### Effect Measure Modification of Differentially Methylated Genes by Newborn Sex

Of the 42 CpGs found to be related to acetaminophen use during pregnancy, 6 probes were found to have significant *P* values for an interaction with newborn sex (*P*_interaction_ < 0.2). Therefore, for these probes, the regression model estimates are presented stratified between male and female newborns (Table 3). For probes cg26893147 located between S100 calcium binding proteins (*S100A7* and *S100A6*), cg00235870 (mapping to BCS1 homolog, *BCS1L*), and cg15551981 located between exostosis like glycosyltransferase 3, *EXTL3*, and Frizzled Class Receptor, *FZD3*, the magnitude of the association between any maternal acetaminophen use during pregnancy and CpG methylation was stronger among females. For probes cg10970399, cg07825336, and cg12944204 corresponding to ubiquitin-specific peptidase 45 (*USP45*), SPARC Related Modular Calcium Binding 2 (*SMOC2*), and Mitochondrial Ribosomal Protein L23 (*MRPL23*), respectively, associations of acetaminophen use with placental methylation were more pronounced among male newborns.

**Table 3:** sex-specific EWAS results of any maternal acetaminophen use during pregnancy among ELGAN singletons (*n* = 286)

		Male newborns (*n* = 151)					Female newborns (*n* = 135)					
		No acetaminophen (*n* = 63)	Acetaminophen (*n* = 88)				No acetaminophen (*n* = 57)	Acetaminophen (*n* = 78)				
CpG probe	Gene(s)^a^	Median	Median	Coefficient^b^	SE^b^^,^^c^	*P* values^b^	Median	Median	Coefficient^b^	SE^b^^,^^c^	*P* values^b^	*P*_interaction_
		*β* value	*β* value				*β* value	*β* value				
cg26893147	*S100A7 ‖ S100A6*	0.854	0.868	0.094	0.054	8.66E–02	0.826	0.856	0.188	0.043	3.27E–05	9.76E–02
cg00235870	*BCS1L*	0.953	0.955	0.054	0.035	1.27E–01	0.952	0.958	0.269	0.034	6.33E–12	1.09E–01
cg10970399	*USP45*	0.007	0.006	–0.258	0.040	3.84E–09	0.007	0.007	–0.171	0.049	8.43E–04	1.41E–01
cg07825336	*SMOC2*	0.963	0.969	0.300	0.076	1.36E–04	0.966	0.969	0.153	0.064	1.86E–02	1.54E–01
cg15551981	*EXTL3* ‖ *FZD3*	0.387	0.353	–0.180	0.040	1.82E–05	0.376	0.368	–0.295	0.039	3.70E–11	7.88E–03
cg12944204	*MRPL23*	0.941	0.946	0.237	0.077	2.50E–03	0.939	0.945	0.132	0.066	4.83E–02	7.86E–02

a Gene or nearest genes as indicated by ‖ for intergenic probes.

b Robust linear regression results of methylation *M* values regressed on any maternal acetaminophen use during pregnancy compared to no use, adjusted for maternal age, race/ethnicity, educational attainment, public health insurance status, cigarette smoke exposure, prepregnancy body mass index, maternal acute illness, maternal chronic illness, maternal NSAID use, parity, gestational age, birth weight, and putative cell type proportions.

c SE, standard error.

### Replication of Genes and CpG Sites Previously Reported to Be Associated with Acetaminophen

A comparison was conducted between the results of the present study and CpG sites that have been previously associated with acetaminophen exposure in blood [22]. Of the 42 differentially methylated sites, there was no overlap. However, 6 of the 29 differentially methylated genes identified in the study overlapped with Gervin et al. (Supplementary Table S1). The genes are potassium voltage-gated channel subfamily E regulatory subunit 3 (*KCNE3*), methionyl-TRNA synthase (*MARS*), mitochondrial ribosomal protein L23 (*MRPL23*), SPARC-related modular calcium binding 2 (*SMOC2*), telomerase reverse transcriptase (*TERT*), and *Zinc Finger Protein 837* (*ZNF837*).

## Discussion

Several studies have demonstrated that acetaminophen has endocrine disrupting activities and may alter neurodevelopment and reproductive development of offspring exposed prenatally [32, 37, 38]. In addition, a recent study has highlighted the relationship between acetaminophen use during pregnancy and CpG methylation in cord blood [22]. The mechanism by which acetaminophen may impact the development of offspring could be multifactorial including effects to the placenta during pregnancy. In the present study, we tested the hypothesis that maternal acetaminophen use during pregnancy would be associated with placental CpG methylation. CpG methylation is an epigenetic mechanism that could underlie adverse placental physiology and impact both fetal development and health outcomes later in life. Placental CpG methylation was compared between women who reported using acetaminophen during pregnancy versus those that did not. Compared to no use, any use of acetaminophen while pregnant was associated with placental methylation levels at 42 CpG sites corresponding to 29 genes and several of these have known functionality in the placenta. Interestingly, several CpG sites displayed sexual dimorphism in their methylation levels where the patterning depended on the sex of the offspring.

The top three probes and their associated genes that were the most significantly associated with maternal acetaminophen use were cg02191312 (*PTGDR*), cg12202498 (*EAF1*), and cg09643313 (*PPP1R9A*). Of note, *PTGDR* is the primary receptor for prostaglandins, a group of physiologically active lipid compounds that are synthesized by cyclooxygenase (COX)-mediated conversion of arachidonic acid [39, 40]. Like most nonsteroidal anti-inflammatory drugs (NSAIDs), acetaminophen acts via inhibition of COX enzyme, thereby inhibiting prostaglandin synthesis [39, 40]. However, unlike NSAIDs, acetaminophen does not have anti-inflammatory properties [41]. Prostaglandin receptors are localized to the amnion, placenta chorion trophoblast and syncytiotrophoblast and they mediate prostaglandin activities during pregnancy [42]. Prostaglandins are involved in several processes including placental blood flow and hormone regulation [43, 44]. The human placenta has been shown to synthesize prostaglandins and it is also permeable to maternal prostaglandins. A progressive increase in prostaglandins by the trophoblast cells has been shown to activate the hypothalamic–pituitary–adrenal axis and has been linked to the rapid increase in fetal growth [45]. Interestingly, abnormal levels of placental prostaglandins have been linked to pregnancy complications such as pre-eclampsia [46]. Our data suggest that the prostaglandin synthesis pathway may be disrupted in the placenta in relation to *in utero* acetaminophen use. *EAF1* functions as an RNA polymerase transcription elongation factor and is highly expressed in several endocrine organs including the uterus, testis, and the placenta [47]. Among its many functions, *EAF1* represses patterning of neuroectoderm and mesoderm during embryogenesis by downregulating the Wnt/β-catenin signaling pathway, a pathway that is essential for placentation and neurodevelopment, and development of female and male reproductive systems [48, 49]. While the role of *PPP1R9A* in the placenta is understudied, it is a protein phosphatase complex considered to be an “epigenetic hotspot on chromosome 7 for ASD” [50]. In neurons, it is involved in maturation of neuronal dendrites and is also implicated in ASD [50–52]. The identified genes from the present study should be pursued in future research to investigate their role in the placenta as it relates to acetaminophen use during pregnancy.

The heterogeneity of the association between acetaminophen use and placenta methylation was assessed by newborn sex and six CpG sites that displayed sex-based differences were identified. Three of these sites displayed significance only in relation to acetaminophen use in male placentas. In contrast, three separate sites displayed significance only in relation to acetaminophen use in female placentas. The probe that displayed the strongest significance in the male-derived placentas maps to ubiquitin specific peptidase (*USP45*, cg10970399). In contrast, the probe that displayed the strongest significance in female-derived placentas corresponds to the mitochondrial chaperon (*BCS1L*, cg00235870). USP family proteins are primarily involved in protein ubiquitination. Protein ubiquitination is essential for function of all eukaryotic cells and in the placenta, ubiquitin proteins in the human cytotrophoblast cells are important for placental development [53]. *BCS1L* is located in the inner mitochondrial membrane and is involved in the assembly of complex III [54]. Interestingly, mutation in *BCS1L* is associated with GRACILE syndrome, a genetic disease characterized by growth and mental retardation. Our results suggest that CpG methylation in the placenta associated with *in utero* acetaminophen exposure may be influenced by infant sex. Interestingly, a sexually dimorphic response to acetaminophen has been observed in both rodents and humans. For example, in mice, females metabolize acetaminophen slowly yet they are more resistant to acetaminophen induced liver toxicity [33]. On the other hand, males metabolize acetaminophen faster than females, yet males are more susceptible to acetaminophen induced liver injuries [33]. Recently, a population study of 2644 mother–infant pairs demonstrated that mothers who used acetaminophen during pregnancy were more likely to have male children with autism spectrum disorders than female children [32]. Although the current study does not provide data that one sex is more susceptible to acetaminophen versus the other, the differences in the strength of CpG methylation association provide further support for the sexually dimorphic paradigm observed with acetaminophen exposure.

To our knowledge, this is the first study to focus on the association between *in utero* acetaminophen exposure and altered epigenetic signatures in the placenta. A recent epigenome-wide association study evaluated acetaminophen in relation to CpG methylation in cord blood [22]. Gervin et al. investigated long-term prenatal acetaminophen exposure (i.e. more than 20 days) among 384 children diagnosed with ADHD and found 6211 CpG sites corresponding to 5038 genes to be differentially methylated between cord blood collected from acetaminophen exposed newborns and controls. When the CpG sites and genes identified in the present study were compared to Gervin et al, a total of six genes were identified that overlapped. The genes were *KCNE3*, *MARS*, *MRPL23*, *SMOC2*, *TERT*, and *ZNF837*. Interestingly, two of these genes, *SMOC2* and *MRPL23*, displayed CpG methylation in the male placenta. Thus, there are some similarities between the identified genes in this study and Gervin et al.

When interpreting the results of this study, several factors should be considered. First, all placentas in the ELGAN cohort were collected from extremely preterm births (i.e. <28 weeks gestation); thus, the findings from this study may not be generalizable to full-term births. Second, while we included a large number of covariates in our models, there may be residual confounding. Notably, confounding by indication bias is a concern because we were lacking information regarding which maternal acute and chronic illnesses prompted acetaminophen use. It is possible that mothers with illnesses or complications took acetaminophen to ameliorate their symptoms; as a result, we cannot rule out the possibility that the epigenetic markers we identified could be reflective of these health conditions rather than acetaminophen exposure through a reverse causal pathway. Third, acetaminophen use was self-reported and may be subject to misclassification bias. Although prior research has found that mothers are typically able to recall whether or not they were exposed to medications during their pregnancy fairly accurately, mothers in this cohort were specifically asked about Tylenol use even though acetaminophen is included in many other products [55]. Fourth, we did not have data on the frequency or dosage of acetaminophen use. A mother who used acetaminophen only once throughout her pregnancy was therefore assumed to be “exposed” in the same way as a mother who took the drug multiple times. Relatedly, we were unable to conduct dose–response analyses. Fifth, our study focuses on CpG methylation in the placenta and does not necessarily represent methylation in all tissues of the fetus. Rather, CpG methylation in the placenta is viewed as a “biological recording” of placental signaling that may affect fetal development and later life outcomes [56]. Placental RNA or proteins from this cohort is not currently available to functionally validate our findings. With this in mind, it is important to note the many strengths of the present analysis. It is the first study to explore the potential impact of maternal acetaminophen use during pregnancy on the placental epigenome and to determine whether significant sex differences existed in the placental epigenome in response to *in utero* acetaminophen exposure.

In summary, acetaminophen exposure *in utero* has been implicated in adverse outcomes in the offspring, but the mechanisms underlying these associations are poorly understood. The major findings from the present study include: (i) prenatal acetaminophen exposure is associated with variation in CpG methylation in the placenta and (ii) the strength of association for certain CpG sites appears to be modified by sex of the offspring. Acetaminophen-dependent CpG methylation sites include genes that play a role in placental physiology. As the understanding of the role of prenatal acetaminophen exposure on fetal development and later health outcomes increases, these data may highlight the role of the placenta epigenome as a mediator of the effects on *in utero* acetaminophen exposure.

## Methods

### Study Population

The ELGAN study is a multicenter cohort designed to evaluate protective and risk factors for brain damage among newborns born prior to 28 weeks of gestation. Participating institutions recruited and enrolled women either shortly after being admitted but before delivering or soon after delivery according to clinical circumstances and/or institutional preference. A total of 1002 singleton newborns were enrolled in the overall ELGAN cohort between 2002 and 2004 across five states (Connecticut, Illinois, Massachusetts, Michigan, and North Carolina). Of these, quantification of placental DNA methylation was confined to a sub-study of 305 singletons who returned for follow-up assessments at 10 years of age and for whom sufficient tissue samples were available. Institutional review boards at each of the 14 participating institutions approved the study procedures, and all mothers provided written informed consent for themselves and their children.

### Assessment of Maternal Medication Use during Pregnancy, Sociodemographics, and Health Status

After delivery, trained research nurses interviewed mothers using structured data collection forms. Mothers were asked to self-report if they had taken Tylenol (acetaminophen) at least once during their pregnancy but prior to hospital admission for delivery. If a mother responded “yes,” she was asked to report what months of pregnancy she initiated and ceased using the drug.

### Maternal Sociodemographics and Health Status

Mothers self-reported their sociodemographic characteristics including their age, race/ethnicity, health insurance type, and educational attainment at the time of delivery. Shortly after the mother’s discharge, the research nurses reviewed the maternal chart using a second structured data collection form in order to abstract the mother’s reproductive history and health conditions, and prescribed medication use.

### Newborn Characteristics

Gestational ages were estimated for most newborns (62%) by dating from fetal ultrasounds conducted prior to the 14th week of pregnancy or according to the dates of embryo retrieval or intrauterine insemination if artificial reproductive technologies were used. If these data were unavailable, gestational age was estimated from later fetal ultrasounds (29%), self-reported last menstrual period (7%), or based on logs from the neonatal intensive care unit (2%). The newborn’s weight in grams was recorded shortly after birth in either the delivery room or upon admission to the neonatal intensive care unit.

### Placental Samples

Placentas were placed in a sterilized basin and biopsied in a sampling room after delivery generally in under 1-hour postpartum. The amnion was pulled back to expose the chorion at the midpoint of the longest distance between the cord insertion and edge of the placental disk. A sample (representing the fetal side of the placenta) of less than 1 g was removed by applying traction to the chorion and underlying trophoblast tissue. The collected specimen was immediately placed in a cryogenic vial and immersed in liquid nitrogen. Samples were frozen and stored at –80°C until shipped to the University of North Carolina at Chapel Hill for processing. There, a 0.2 g subsection of the placental tissue was cut from the frozen biopsy and washed with sterile 1× phosphate-buffered saline to remove any remaining blood. Tissues were lysed by homogenizing the subsections with β-mercaptoethanol in Buffer RLT (Qiagen, Valencia CA).

### Placental DNA Methylation

DNA sequences >18 nucleotides long were isolated using ALLPrep DNA/RNA/miRNA Universal Kit (Qiagen). Extracted DNA samples were then shipped on dry ice to Wayne State University where bisulfite conversion was performed using the EZ DNA Methylation Kit (Zymo Research, Irvine, CA). The Illumina Infinium MethylationEPIC BeadChip (Illumina, San Diego, CA) was then used to profile methylation status at more than 850 000 CpG sites across the genome. Average methylation values (*β* values) were computed to represent the ratio of methylated to unmethylated signal intensities.

A total of 810 probes were removed due to intensity values that fell below background levels (detection *P* > 0.01), probes located on X or Y chromosomes (*n* = 19 600), non-CpG probes (*n* = 2839), and probes previously identified as containing single nucleotide polymorphisms (*n* = 12 224) or cross-reactive (*n* = 40 492) yielding a total of 790 677 probes for analyses *β* values were background corrected using the normal-exponential out-of-band (*noob*) correction method and normalized with functional normalization [57]. To evaluate batch effects, a principal component analysis (PCA) was performed. Plate was identified as a significant source of variation; thus, we corrected the data using the *ComBat* package [58]. PCA was then repeated on the corrected data and the results suggested this bias was sufficiently removed (Supplementary Fig. S1). Methylation *β* values at each CpG site were then logit-transformed to obtain *M* values [log_2_(*β*/(1 − *β*)], as *M* values are considered more statistically valid for the differential analysis of methylation levels [59].

### Statistical Analyses

Separate robust linear regressions were fit to model the relationships between CpG methylation *M* values and maternal acetaminophen use during pregnancy at each CpG loci. All models were adjusted for maternal age, race/ethnicity, educational attainment, public health insurance status, cigarette smoke exposure, prepregnancy body mass index, maternal acute illness, maternal chronic illness, maternal NSAID use, parity, newborn sex, gestational age, and birth weight. These variables were selected *a priori* as they represent important antecedents of acetaminophen use in pregnancy and/or have been found to influence placental methylation signatures. Further adjustments were made for cellular composition by using surrogate variables from the *RefFreeEWAS* R package [60, 61]. Maternal age (years), prepregnancy body mass index (kg/m^2^), parity (number of deliveries), gestational age (weeks), and birth weight (grams) were modeled as continuous covariates. Maternal race/ethnicity (non-Hispanic white, non-Hispanic black, non-Hispanic other, Hispanic), educational attainment (high school diploma or less, at least some college, college degree or greater), public health insurance status (yes or no), cigarette smoke exposure (yes or no), acute illness (yes or no), chronic illness (yes or no), NSAID use (yes or no), and newborn sex (male or female) were parameterized using dummy variables. Public health insurance, specifically referred to Medicaid coverage, and educational attainment were considered proxies for socioeconomic status. Cigarette smoke exposure was defined as self-reported active smoking or secondhand exposure during pregnancy. Fever, upper respiratory infection, urinary tract infection, bronchitis, or antibiotic use while pregnant but prior to delivery was considered indicative of acute illnesses whereas chronic illnesses included a history diabetes or antidiabetes medication use, hypertension or antihypertensive medication use, thyroid alterations or thyroid medication use, or renal conditions. An epigenome-wide association study (EWAS) was performed to assess the association of any acetaminophen use during pregnancy with CpG methylation. Newborns born to mothers who reported no use of acetaminophen while pregnant served as the reference group. Genomic inflation was examined via the genomic inflation factor (*λ*) and by making quantile–quantile (Q–Q) plots. Any inflation of test statistics and corresponding effect sizes, standard errors, and *P* values was corrected using a Bayesian method implemented within the R package *bacon* [36]. To account for multiple testing, we controlled for the false discovery rate with *q*-values <0.05 considered statistically significant [62]. Finally, for all significant CpGs (*q* < 0.05), we tested whether the association with acetaminophen use differed between male and female newborns by adding a multiplicative interaction term to the models. All statistical analyses were conducted using R version 3.5.1 (R Core Team 2018). Annotation of the top differentially methylated CpGs was performed using the Infinium MethylationEPIC manifest file (version 1.0) from Illumina.

## Supplementary Material

dvz010_Supplementary_Data
