# Acetaminophen use during pregnancy and DNA methylation in the placenta of the extremely low gestational age newborn (ELGAN) cohort

**DOI:** 10.1093/eep/dvaa006

**Published:** 2020-05-06

**Authors:** Kezia A Addo, Catherine Bulka, Radhika Dhingra, Hudson P Santos, Lisa Smeester, T Michael O’Shea, Rebecca C Fry

*Environmental Epigenetics 2020*

doi: 10.1093/eep/dvz010

It has come to the attention of the authors that an error occurred during sample processing. Placental DNA samples on 96-well plate were misoriented to their matching barcodes. This resulted in a mismatch of methylation and phenotype data. These errors influenced results published in *Environmental Epigenetics*: Kezia A Addo, Catherine Bulka, Radhika Dhingra, Hudson P. Santos Jr, Lisa Smeester, T. Michael O’Shea and Rebecca C. Fry. *Acetaminophen use during pregnancy and DNA methylation in the placenta of the extremely low gestational age newborn (ELGAN) cohort*. Once correctly aligned, we identified a small number of samples for which the reported infant sex and methylation-predicted sex (using the *minfi* R package) differed. Correction for these errors resulted in changes in beta estimates, p-values, and the CpG probes identified as significant.

For this re-analysis, we excluded five placentas with an apparent sex mismatch (two infants reported to be female and three reported to be male), decreasing our sample size from n=286 to n=281. Previously, a total of 42 CpG sites were identified as differentially methylated comparing placental tissues from mothers who reported acetaminophen use during pregnancy and those who did not (FDR<0.05). Upon re-analysis, 17 (40.5%) of the previously identified 42 CpG sites remained significant. We identified an additional seven FDR-significant probes for a total of 24 FDR-significant hits. With respect to interaction by fetal sex, we previously identified six CpGs (P_interaction_ <0.2); none of these remained significant upon re-analysis. However, we identified nine different probes that showed evidence of interaction by fetal sex (P_interaction_ <0.2).

