# Supplementary material for: Acetaminophen use during pregnancy and DNA methylation in the placenta of the extremely low gestational age newborn (ELGAN) cohort
Source: Environ Epigenet. 2019 Aug 6;5(2):dvz010. doi: 10.1093/eep/dvz010 (PMC6682751; doi:10.1093/eep/dvz010)
Supplement: dvz010_Supplementary_Data [file dvz010_supplementary_data.zip › Supplemental Material revision #2-clean.docx]

**Figure S1.** Box-whisker plots of batch effects in principal component 1 before and after *ComBat* adjustment.

**
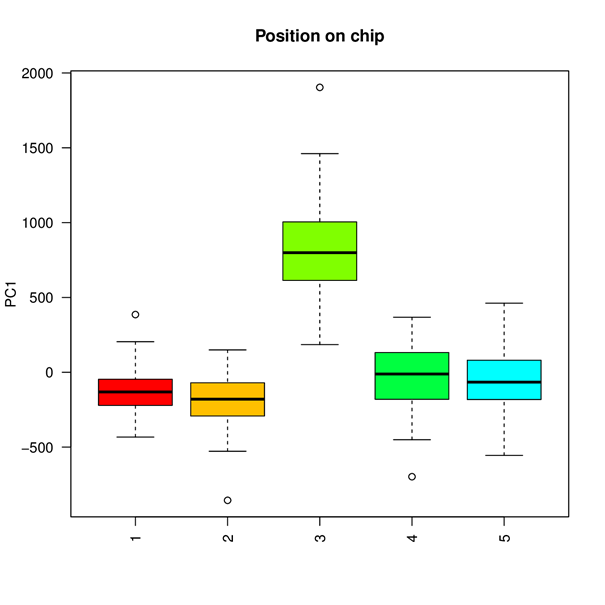

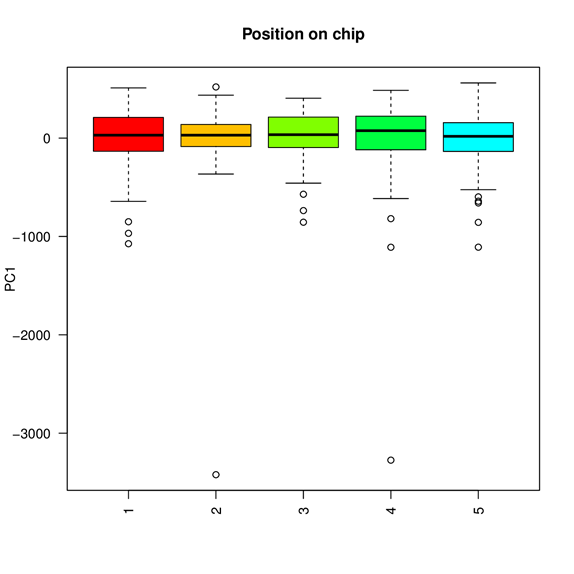
**

**Figure S2.** Quantile-quantile (Q-Q) plot for the association between maternal acetaminophen use during pregnancy and CpG methylation, before and after *bacon* correction for genomic inflation.


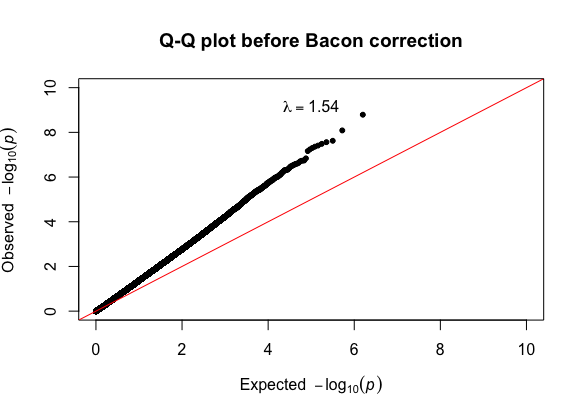

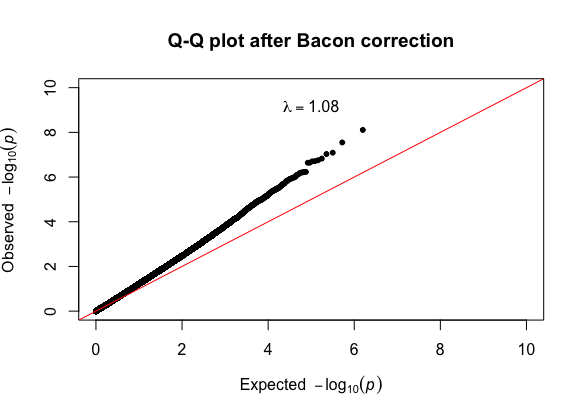


**Figure S3.** Manhattan plot displaying epigenome-wide association results for any maternal acetaminophen use during pregnancy compared to no use. The models adjusted for maternal age, race/ethnicity, educational attainment, public health insurance status, cigarette smoke exposure, pre-pregnancy body mass index, maternal acute illness, maternal chronic illness, maternal nonsteroidal anti-inflammatory drug use, parity, newborn sex, gestational age, birth weight, and putative cell type proportions. The blue line represents the FDR (q < 0.05). The red line represents a Bonferroni correction of 6.3 x 10^-8^.

**
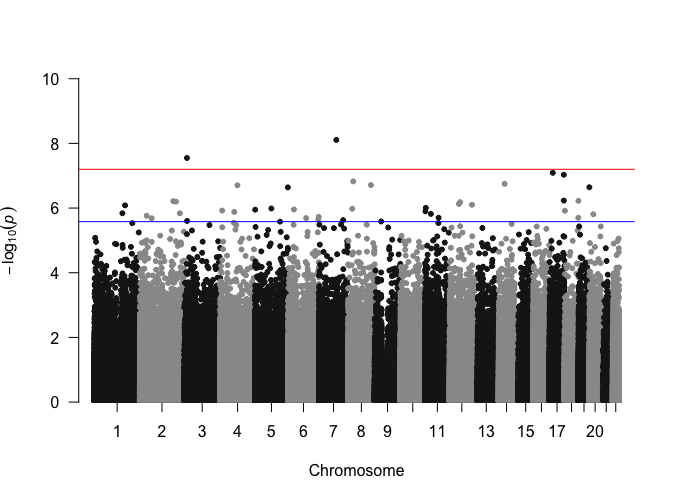
**

**Figure S4.** Beeswarm plots of methylation β values for the 42 CpGs differentially methylated in relation to any maternal acetaminophen use during pregnancy.


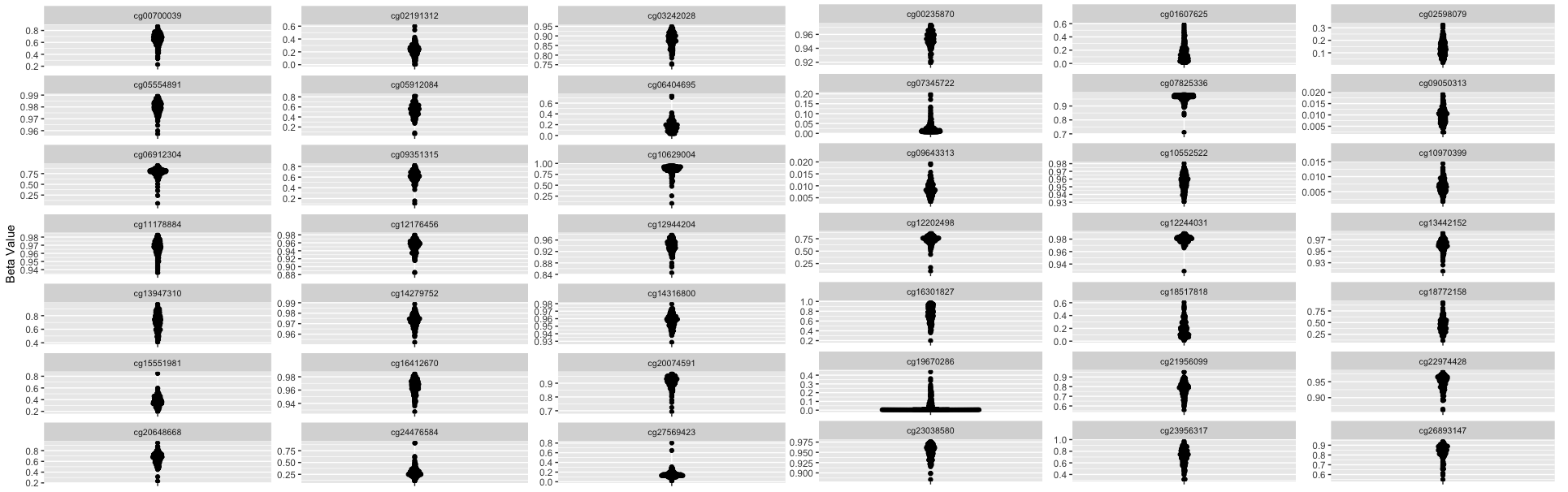


**Figure S5.** Gene probe site distribution and location for all probes contained on the Illumina Human Methylation 850 Bead Chip (pink) and differentially methylated cites between placentas exposed to acetaminophen and unexposed.

**
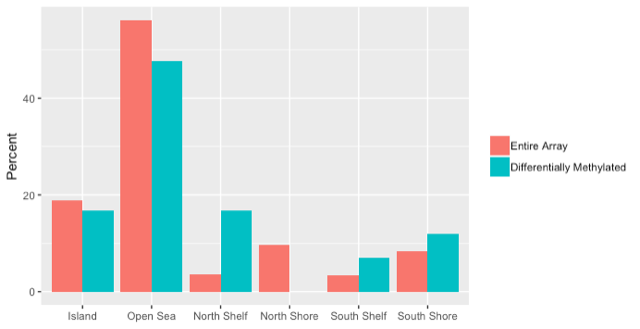
**

**Supplementary Table 1:** Overlap of genes that were differentially methylated in the present study and also in Gervin *et al.* 2017.

| CpG Probe | Gene | Chr:Position | P value |
| --- | --- | --- | --- |
| cg21956099 | *TERT* | 5: 1278018 | 1.12E-06 |
| cg20074591 | *KCNE3* | 11: 74179549 | 4.41E-02 |
| cg11178884 | *MARS* | 12: 57906632 | 3.45E-02 |
| cg07825336 | *SMOC2* | 6: 169039836 | 4.41E-02 |
| cg24476584 | *ZNF837* | 19: 58883830 | 1.81E-02 |
| cg12944204 | *MRPL23* | 11: 1971969 | 3.83E-02 |
